# Commentary: Perioperative blood loss is a risk factor for postoperative delirium in geriatric hip fracture patients: a retrospective study

**DOI:** 10.3389/fmed.2026.1753996

**Published:** 2026-04-23

**Authors:** Samuel Singha, Benjamin Howell Lole Harris, Michael Barry Fertleman, Louis John Koizia

**Affiliations:** 1 Imperial College Healthcare NHS Trust, London, United Kingdom; 2 Cutrale Perioperative and Ageing Group, Department of Bioengineering, Imperial College London, London, United Kingdom; 3 St. Catherine's College, University of Oxford, Oxford, United Kingdom; 4 Department of Oncology, University of Oxford, Oxford, United Kingdom; 5 Division of Medicine, Institute for Liver and Digestive Health, University College London, London, United Kingdom; 6 Department of Psychosocial Rehabilitation, Medical University of Lodz, Lodz, Poland; 7 Collaborating Centre for Values-based Practice, St Catherine's College, Oxford, United Kingdom

**Keywords:** anemia, blood loss, delirium, hip fracture, perioperative

We commend Deng et al. (1) for providing a timely and clinically relevant analysis of postoperative delirium (POD) in older adults undergoing hip fracture surgery. Prior work has long suggested an association between intraoperative physiological disturbance and delirium risk, but often lacked the granularity achieved here (2).

The demonstration of a relationship between perioperative blood loss and POD risk is striking and warrants careful clinical interpretation. However, additional nuance is needed before blood loss can be viewed as an independent causal factor rather than a marker of underlying vulnerability. Patients who bleed more during hip fracture surgery are often those inherently more susceptible to delirium. In addition, several determinants of increased intraoperative bleeding are also well-established risk factors for delirium (3).

Pre-existing anticoagulation and antiplatelet therapy represent an important omission in this study. Many older adults with atrial fibrillation, coronary artery disease or peripheral vascular disease receive anticoagulation or antiplatelet therapy, all of which increase perioperative bleeding risk. These therapies also tend to cluster with conditions characterized by impaired cerebral autoregulation and vascular insufficiency, both contributors to delirium vulnerability (4). Without accounting for these medications, the association between blood loss and POD may instead reflect these underlying factors rather than blood loss itself.

Frailty, sarcopenia, malnutrition and reduced physiological reserve also increase both bleeding tendency and delirium susceptibility. Frail individuals have reduced circulating volume, diminished haemostatic capacity and heightened inflammatory responses, all of which are strongly predictive of POD (3, 5, 6). However, no frailty index, cognitive baseline, functional status or nutritional marker was included. Similarly, the absence of fracture type or surgical approach introduces residual confounding, as unstable fractures are associated with greater blood loss and occur in individuals with her co-morbidity burden.

Another important consideration is transfusion. The method used to calculate total blood loss incorporates transfused volume, yet transfusion was not analyzed independently. Prior studies demonstrate that transfusion is itself associated with POD through inflammatory activation and endothelial dysfunction (3, 7). Without separating the effects of anemia, tissue hypoxia and transfusion-related mechanisms, it is difficult to determine the true mediator of delirium risk. This reflects a broader methodological challenge in clinical research, where composite or indirect measures may obscure the underlying biological signal unless carefully disentangled and validated (8, 9).

Despite these limitations, Deng et al. are to be congratulated for highlighting a clinically important perioperative factor. Their work reinforces the close link between intraoperative physiology and postoperative cognitive outcomes and provides a useful foundation for future studies incorporating frailty, anticoagulation status, fracture complexity and transfusion dynamics. Overall, this study adds meaningful evidence to support haemostatic stability as part of delirium prevention strategies in hip fracture care. The authors should be commended for advancing this important dialogue in orthogeriatric surgery.
